# Supplementary material for: Epidemiological patterns of motorcycle-related injuries in Cameroon: A comparative analysis of motorcycle users and pedestrians
Source: PLOS Glob Public Health. 2026 Apr 20;6(4):e0005816. doi: 10.1371/journal.pgph.0005816 (PMC13094955; doi:10.1371/journal.pgph.0005816)
Supplement: Attachment — Submitted filename: EPID Responses to editorial and reviewers comments.docx [file pgph.0005816.s002.docx]

**Responses to editorial and reviewers comments**

We thank the Editor and Reviewers for their thoughtful and constructive comments. We have carefully revised the manuscript and addressed each comment below.

1. Please ensure that your Ethics Statement is available in its entirety at the beginning of your Methods section, under a subheading 'Ethics Statement'.

**Author’s response: Thank you. The ethics statement has been placed as recommended at the start of the methods section.**

2. We note that your Data Availability Statement is currently as follows: All relevant data are within the manuscript.
 
Please confirm at this time whether or not your submission contains all raw data required to replicate the results of your study. Authors must share the “minimal data set” for their submission. PLOS defines the minimal data set to consist of the data required to replicate all study findings reported in the article, as well as related metadata and methods (<https://journals.plos.org/plosone/s/data-availability#loc-minimal-data-set-definition>).
 
 For example, authors should submit the following data:
 
 - The values behind the means, standard deviations and other measures reported;
 - The values used to build graphs;
 - The points extracted from images for analysis.
 
 Authors do not need to submit their entire data set if only a portion of the data was used in the reported study.
 
 If your submission does not contain these data, please either upload them as Supporting Information files or deposit them to a stable, public repository and provide us with the relevant URLs, DOIs, or accession numbers. For a list of recommended repositories, please see <https://journals.plos.org/plosone/s/recommended-repositories>.
 
**Author’s response: Thank you. All supporting data has been included in the attached supporting information file.**

Please review your reference list to ensure that it is complete and correct. If you have cited papers that have been retracted, please include the rationale for doing so in the manuscript text, or remove these references and replace them with relevant current references. Any changes to the reference list should be mentioned in the rebuttal letter that accompanies your revised manuscript. If you need to cite a retracted article, indicate the article’s retracted status in the References list and also include a citation and full reference for the retraction notice.
**Author’s response: Thank you very much. We reviewed the reference list to ensure completeness and accuracy. No retracted articles are cited.**

**Reviewers' comments:**

**Reviewer #1:**

This manuscript presents a comparative analysis of motorcycle-related injuries among motorcycle users and pedestrians using data from the Cameroon Trauma Registry. While the study does not introduce a novel methodological approach or previously unreported risk factors, it provides multicentre, prospectively collected data from a low- and middle-income country where such evidence remains limited. In that sense, it represents a useful and necessary contribution to the literature.
**Author’s response: Thank you for the complement**

The strengths of the study lie in its relatively large sample size, inclusion of multiple hospitals across different regions, standardized data collection through a trauma registry, and clear stratification of outcomes between motorcycle users and pedestrians. The descriptive epidemiology is sound and confirms patterns reported from other studies from LMICs - low helmet use, alcohol involvement, high hospitalization rates, and substantial post-injury disability. Importantly, the manuscript highlights the often-underreported burden among pedestrians, particularly older women, which adds contextual value for policy discussions.

That said, the findings are largely confirmatory rather than novel. The discussion occasionally reiterates well-established recommendations (helmet enforcement, alcohol deterrence, trauma system strengthening) without offering substantially new insights into implementation or prioritization.

Overall, despite limited novelty, this manuscript provides much needed epidemiological data from an LMIC setting and fills an important evidence gap. I believe it is suitable for publication, as it adds a valuable data point to the global road injury literature and can inform context-specific prevention and trauma system planning.
**Author’s response: Thank you. We greatly appreciate your comments.**

**Reviewer #2:**

Thank you for this well done job.

Only a few minor comments arise.

1) The sequence of reporting of the ethical approval presents a subtle focus on the California ethical approval for a Cameroonian registry research. Authors should consider flipping this- both Cameroonian approvals should be placed first in sequence, and then California approvals.
**Author’s response: Thank you. We have readjusted the sequence of ethical approval so that local approval come first on the list.**

2) Table 1 conflates categories which should be clarified.
i) Divorced can be single or living with partner.
ii) Married can be living with partner.
iii) Widowed can be single or living with partner.
Consider collapsing categories as appropriate. Footnotes would be helpful for clarity
**Author’s response: Thank you. This has been adjusted accordingly**

iv) Non-employed versus unemployed? Footnotes would be helpful for clarity
**Author’s response: Thank you. We have clarified the distinction between non-employed and unemployed in a table footnote.**

3) Clarify what "domestic activities" lead to motorcycle crashes, perhaps with a footnote to the table with examples. This is not intuitive that domestic activities in the house would lead to motorcycle crashes.
**Author’s response: Thank you. We have added a footnote explaining that domestic activities refer to household-related travel such as market visits and errands.**

4) The term perpetrator in Table 2 (“Alcohol use among perpetuator”) is almost vilifying. Can this be replaced? Again if perpetuator means the rider who “crashed” on the pedestrian or pinion/passenger, can this individual not also suffer injury and be a patient?

**Author’s response: Thank you. We have replaced “perpetuator” to “motorcycle driver” as they were the ones that injured the pedestrians. Yes, they can also be patients if injured and arrived at the hospital.**

An finally,
5) So that tables can be standalone, please ensure that abbreviations are embedded in them.

**Author’s response: Thank you. All abbreviations have now been embedded as footnotes under the tables.**

All underlying data are not within the manuscript- results are included in some places, but not underlying data. For instance- for Abbreviated injury severity score- we do not have the data of the scores, but of the category. For several others, we have counts and percentages, but not underlying data that we can use to cross check analyses. If there are caveats with sharing registry data, kindly note them rather than stating that all data is within the manuscript.
**Author’s response: Thank you. Supporting data are now presented in the supporting information file.**
